# Calculating the number of undetected active SARS-CoV-2 infections from results of population-wide antigen tests

**DOI:** 10.25122/jml-2021-0243

**Published:** 2021

**Authors:** Fabian Standl, Bernd Kowall, Anna Katharina Frost, Bastian Brune, Marcus Brinkmann, Marcel Dudda, Florian Oesterling, Philipp Jansen, Karl-Heinz Jöckel, Andreas Stang

**Affiliations:** 1. Institute for Medical Informatics, Biometry and Epidemiology, University Hospital Essen, Essen, Germany; 2. Medical Direction Communal Emergency Service of the City Essen, Essen, Germany; 3. Clinic for Trauma, Hand and Reconstructive Surgery, University Hospital Essen, Essen, Germany; 4. Center for Clinical Trials Essen, University Hospital Essen, Essen, Germany; 5. Federal Cancer Registration gGmbH, Bochum, Germany; 6. Clinic for Dermatology, University Hospital Essen, Essen, Germany; 7. Department of Epidemiology, Boston University, Boston, United States of America

**Keywords:** pandemic, corona, antigen tests, dark figure, SARS-CoV-2

## Abstract

Current European research estimates the number of undetected active SARS-CoV-2 infections (dark figure) to be two- to 130-fold the number of detected cases. We revisited the population-wide antigen tests in Slovakia and South Tyrol and calculated the dark figure of active cases in the vulnerable populations and the number of undetected active cases per detected active case at the time of the population-wide tests. Our analysis follows three steps: using the sensitivities and specificities of the used antigen tests, we first calculated the number of test-positive individuals and the proportion of actual positives in those who participated in the antigen tests. We then calculated the dark figure in the total population of Slovakia and South Tyrol, respectively. Finally, we calculated the ratio of the dark figure in the vulnerable population to the number of newly detected infections through PCR tests. Per one positive PCR result, another 0.15 to 0.71 cases must be added in South Tyrol and 0.01 to 1.25 cases in Slovakia. The dark figure was in both countries lower than assumed by earlier studies.

## Introduction

Slovakia and South Tyrol conducted population-wide antigen tests to identify individuals currently infected with “severe acute respiratory syndrome coronavirus type 2” (active cases), internationally abbreviated as SARS-CoV-2, and determine the number of undetected infections in the respective population. In Slovakia, the antigen tests were conducted on October 31^st^ and November 1^st^, 2020. From a total population of 5,460,550 inhabitants, 3,625,332 (66.4%) persons were tested and 38,359 (1.1%) had a positive test result [1]. In South Tyrol, from a total population of 536,667 inhabitants, 361,781 (67.4%) persons were tested, and 3,615 (1.0%) had a positive test result. The antigen tests in South Tyrol were predominantly conducted between November 20^th^ and 22^nd^. However, the official counting included antigen test results between November 18^th^ and 25^th^ [2]. The objective of this work was to calculate the number of undetected active cases, commonly known as “dark figure”, from the results of the population-wide antigen tests. In contrast to previous European studies [3–11], our calculations are based on the diagnostic criteria of the antigen tests and therefore take false-positive and false-negative results into account. This leads to a more precise calculation of the dark figure.

## Material and Methods

Sensitivity and specificity of the used antigen tests: Slovakia used the antigen tests “Biocredit COVID-19 Ag” and “Standard Q COVID-19 Ag Test”. In South Tyrol, the antigen tests “Standard Q COVID-19 Ag Test” and “Panbio COVID-19 Ag Rapid Test Device” were used [12, 13]. For each antigen test, sensitivity and specificity were reported for various cycle threshold (Ct) values. As individuals might only hold a low viral load, we used the diagnostic criteria for Ct values of ≤33. For the Panbio test, the respective values were not available, so we used the specifications for Ct ≤34. In contrast to the other tests, there were two survey places mentioned for the Standard Q Test (Brazil and Germany, meanwhile and after our analysis, the corresponding values for Switzerland were published as well) with different values for sensitivity and specificity – we considered both places in our calculations. This resulted in nine combinations of sensitivity and specificity (in each case a point-estimate and two 95% confidence limits) per test and place – e.g., the upper limit of sensitivity combined with the upper limit of specificity, the upper limit of sensitivity combined with a point estimate of specificity etc. The sensitivity and specificity and the corresponding confidence interval for each test were at the time of the population-wide antigen tests: 1. Biocredit 82.5% (73.7% 88.8%) and 98.95% (97.2% 99.6%), 2. Standard Q 87.8% (74.5% 94.7%) and 99.3% (98.6% 99.6%) for Germany respectively 91.9% (84.9% 95.9%) and 97.6% (95.2% 98.6%) for Brazil, 3. Panbio 79.6% (67.1% 98.3%) and 100% (98.7% 100%) [14–16]. It was not known in what proportion the individual tests were used in the population-wide tests. Therefore, we calculated the population-wide usage of each test, knowing that the true value of the dark figure will be in between the population-wide usage of the test with the worse and the test with the better diagnostic criteria.

### The three steps of the analysis

First – The number of test-positive Tp cases is the sum of correct and false positives that can be mathematically expressed as:



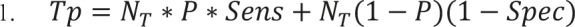



N_T_ is the respective population size (here the population tested with antigen tests), P is the proportion of true positives in the population (here the share of active cases in the antigen test tested population), Sens is the sensitivity, and Spec the specificity of the respective test. This formula can be resolved for P to estimate the proportion of active cases in the respective antigen tested population with the formula below:



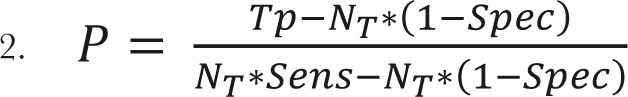



P becomes negative if N_T_*(1-Spec) is higher than the number of test-positive persons. We excluded negative values from further analysis and reporting, as negative values would indicate that the tests would result in more false-positive than correct positive results.

Second – The dark figure was calculated with the formula below:



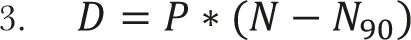



D is the calculated dark figure for the specifications used in the first formula. As mentioned in the introduction, Slovakia tested 66.4% of its population with antigen tests, and 1.1% of the tested population had a positive result. South Tyrol tested 67.4% of its population with antigen tests, and 1.0% of the tested population had a positive result. We assumed P to be the same in N and N_T_. To obtain the dark figure for the total population, P must be multiplied with the total population N. This would, however, include individuals who were already identified with a “polymerase chain reaction” (PCR) test or who have just recovered from an infection, and are immune now. To forestall this selection bias, the responsible health authorities that conducted the antigen tests excluded all individuals N_90_ from the antigen tests who had a positive PCR result within 90 days prior to the population-wide tests [13, 17]. In Slovakia, N_90_ was 55,327, and in South Tyrol N_90_ was 15,293; these numbers were extracted from the respective official national and regional dashboards [18, 19]. The total population N minus N_90_, the amount of PCR identified cases within the 90 days before the population-wide tests can be understood as the vulnerable population. This vulnerable population consists of persons who had not yet been infected with SARS-CoV-2 and persons who have had an infection but are vulnerable again for infection.

Third – To estimate the dark figure per detected active infection, we calculated the ratio of the dark figure D, in the respective N minus N_90_ population, to the number of newly detected infections, identified with a PCR test, within 20 days before the population-wide antigen tests, because: “The median duration for SARS-CoV-2 carrying was 20 days (6 to 50 days) with a P25 of 16 days, and a P75 of 28 days” [20]. This means that we compared the number of PCR detected active infections with the number of usually undetected active infections identified with the population-wide antigen tests. The number of PCR detected cases was 38,867 in Slovakia and 10,917 for South Tyrol. The corresponding data were extracted from the respective official national and regional dashboards [18, 19].

## Results

The results of our calculations are displayed in Table 1 and show that the ratio of undetected active cases per detected active case is considerably lower in South Tyrol than in Slovakia. The Standard Q Test, used in both regions, detected 0.53 to 1.24 undetected cases per detected active case in Slovakia. In South Tyrol, the corresponding values were 0.15 to 0.39. Overall, South Tyrol still had fewer undetected cases per detected active case. Per one positive PCR result, another 0.15 to 0.71 cases must be added in South Tyrol and 0.01 to 1.25 cases in Slovakia.

**Table 1.** Diagnostic criteria of the antigen tests and estimation of the number of undetected active cases (dark figure) per detected active case.

**Country/Region**	**Antigen test**	**Sensitivity in %**	**Specificity in %**	**Adjusted share of positive antigen tests in the tested population (P)**	**Dark figure of active cases in the vulnerable population (D)**	**Undetected active cases per detected active case**
**Slovakia**	**Biocredit**	82.50	98.95	9.92*10^-5^	536	0.01
		82.50	99.60	8.02*10^-3^	43,326	1.11
		73.70	98.95	1.11*10^-4^	601	0.02
		73.70	99.60	8.98*10^-3^	48,528	1.25
		88.80	98.95	9.21*10^-5^	498	0.01
		88.80	99.60	7.44*10^-3^	40,238	1.04
	**Standard Q**	87.80	99.30	4.11*10^-3^	22,222	0.57
		87.80	99.60	8.02*10^-3^	43,326	1.11
		74.50	99.30	4.85*10^-3^	26,226	0.67
		74.50	99.60	8.88*10^-3^	48,004	1.24
		94.70	99.30	3.81*10^-3^	20,591	0.53
		94.70	99.60	6.98*10^-3^	37,721	0.97
**South Tyrol**	**Panbio**	79.60	100.00	1.26*10^-2^	6,545	0.60
		67.10	100.00	1.49*10^-2^	7,764	0.71
		98.30	100.00	1.02*10^-2^	5,300	0.49
	**Standard Q**	87.80	99.30	3.44*10^-3^	1,791	0.16
		87.80	99.60	7.30*10^-3^	3,805	0.35
		74.50	99.30	4.05*10^-3^	2,114	0.19
		74.50	99.60	8.09*10^-3^	4,216	0.39
		94.70	99.30	3.18*10^-3^	1,660	0.15
		94.70	99.60	6.35*10^-3^	3,313	0.30

To manually calculate the table from left to right for each line, please use the respective share of sensitivity and specificity. The amount of test-positive TP individuals was 38,359 in Slovakia and 3,615 in South Tyrol. The antigen tested population size N_T_ was 3,625,332 in Slovakia and 361,781 in South Tyrol. Subsequently, the adjusted share of positive antigen tests in the tested population P can be calculated using the formula below. To calculate the dark figure of active cases in the vulnerable population D, please use the following specifications and formula below: P as calculated in the previous step, the total population size N 5,460,550 was for Slovakia and 536,667 for South Tyrol. The number of people for N_90_, persons who had a positive PCR result in the 90 days before the antigen tests – and were therefore excluded from the antigen tests by the respective health authorities – was 55,327 in Slovakia and 15,293 in South Tyrol. To calculate the undetected active cases per detected active case D, from the previous step, must be divided by the sum of PCR positive cases in the 20 days before the antigen test. For Slovakia, the corresponding sum is 38,867, and for South Tyrol, 10,917. 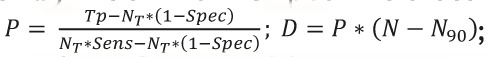 Please note: P becomes negative when the estimated number of false-positive individuals is higher than the number of test-positive persons. We excluded negative values from further analysis and reporting, as negative values would indicate that the tests would result in more false-positive than correct positive results.

As mentioned above, Slovakia tested 3,625,332 (66.4%) of its 5,460,550 inhabitants. 38,359 (1.1%) of the tested population had a positive test result, and 55,327 persons were excluded from the antigen tests by the health authorities as the individuals had a positive PCR result in the 90 days prior to the antigen tests. If 1.1% of the vulnerable population had been tested positive, this would have been about 59,457 persons. According to our calculations, the dark figure’s range in Slovakia was 498 up to 48,528 individuals. In other words, a maximum of 48,528 individuals would have been identified as true positive with the used antigen tests and, at least, about 11,000 persons would have been a false positive. Therefore, the dark figure in the vulnerable population was between 0.0% and 0.9%.

For South Tyrol, the corresponding values are: 361,781 (67.4%) persons out of a total population of 536,667 inhabitants were tested, and 3,615 (1.0%) individuals had a positive antigen test result. 15,293 persons were excluded from the antigen tests by the health authorities as the individuals had a positive PCR result 90 days prior to the antigen tests. If 1.0% of the vulnerable population had been tested positive, this would have been about 5,213 persons. Our calculations resulted in 1,660 up to 7,764 true positives with the antigen tests used. Therefore, the dark figure in the vulnerable population in South Tyrol was between 0.3% and 1.5% of the vulnerable population.

## Discussion

So far, it was expected that a high number of SARS-CoV-2 infections were undetected. Previous European studies based their results on statistical modeling [5, 7–9, 11], serological testing in study participants [3, 6, 10], and the application of study results from a third country to their own [4]. The authors report two to 130 undetected cases per detected case for Austria, Germany, Italy, Spain, and the United Kingdom [3, 4, 6–11], estimates we cannot confirm. According to our calculations, 0.01 to 1.25 cases (Slovakia) and 0.15 to 0.71 (South Tyrol) cases per one positive PCR result must be added – the latter is in line with the retrospective analysis of the COVID-19 pandemic in Italy by Fochesato and colleagues [5]. At the same time, the antigen tests that took place in Slovakia, 21,477 PCR tests were performed additionally, and 4,165 (19.4%) of these were positive. The same was applied in South Tyrol, 21,210 PCR tests were performed, and 3,857 (18.2%) were positive. These positive rates were considerably higher, as they were event-related than the 1.1% and 1.0% reported by the antigen tests, respectively. Therefore, at least for Slovakia and South Tyrol, it can be assumed that the detection mechanisms in place worked well and may have been improved with antigen tests. Calculating the share of fatal infections from the assumed number of total infections, lethality is higher as the total number of infections is lower due to a smaller amount of undetected cases [21]. Our calculations show that the number of undetected cases is lower in Slovakia and South Tyrol than what could be expected from current European research. Evidently, this results in a higher share of fatal cases from the number of total infections and leads to fewer infections but an increased lethality.

It is a limitation that the proportions of the tests used in the two population-wide antigen tests are unknown to the scientific community – precise numbers would increase the precision of the calculations. In addition, the sensitivity and specificity of the antigen tests in the field application in Slovakia and South Tyrol are unknown. Variations of sensitivity and specificity may occur due to differences in testing procedures, personnel training, and other deviations. Moreover, we do not know the impact of the fact that willingness to participate in antigen testing may have varied between age groups; this is unclear, especially for children. Thus, the age distribution in the participants of the antigen tests may differ from the age distribution in the whole population. Finally, the assumption of P to be the same in N and N_T_ is a limitation – although it is likely to be correct, we cannot prove nor deny it. The strength of our calculations is that there is no need for a stochastic model that needs a high dark figure to explain the observed PCR cases. Our calculations that use real-world data and real-world estimates show a decreased number of undetected cases, compared to current literature, and are in line with the observation that the SARS-CoV-2 pandemic has so far been less dynamic than influenza pandemics [22]. To better estimate the dark figure, official statistics should report the amount of false-positive cases in antigen and PCR tests. However, a gold standard assay is missing so far. As suggested, to reduce the number of false positives, test results need to be interpreted against the background of the individual pretest probability [23]. Mahase went one step further and criticized that the only differential diagnostic criteria to diagnose COVID-19 was the positivity of a PCR test. No other clinical features or symptoms were needed for the diagnosis [24]. Stang *et al.* recently demonstrated that a positive SARS-CoV-2 PCR result is not sufficient to be interpreted for COVID-19 diagnosis. Above this, it is demonstrated that asymptomatic individuals have a considerably higher Ct value than symptomatic persons [25]. Again, the lethality of COVID-19 increases when the diagnosis of the illness is based on a more differentiated, clinical image. The dark figure shrinks again as fewer cases are covered by a more differential diagnostic definition. Against the background of the considerable huge amount of false-positive antigen test results in Slovakia, which caused quarantine for truly uninfected persons – an ethical and legal dilemma, a positive antigen test result should always be verified with a PCR test. Quarantine should only be imposed on infectious persons.

## Conclusion

The results of our calculations indicate that the number of undetected SARS-CoV-2 cases in European studies is frequently overestimated. Calculating the dark figure from the results of population-wide antigen tests by using the sensitivity and specificity of the antigen tests used, delivers reliable results for the true number of undetected SARS-CoV-2 infections in a population. Based on our calculations the true number of undetected SARS-CoV-2 cases is lower than what was expected so far, therefore lethality of SARS-CoV-2 is higher than what was expected by current research.

## Acknowledgements

### Conflict of interest

The authors declare that there is no conflict of interest.

### Authorship

FS, BK, BB, MB PJ, KHJ and AS made substantial contributions to the conception and design of the work. FS, BK, AKF, BB, MB, FO, PJ, KHJ, AS contributed to acquisition, analysis, and interpretation of data for the work, drafting and critically revising the work, and final approval. All authors agree to be accountable for all aspects of the work.
